# The Laminin Receptors Basal Cell Adhesion Molecule/Lutheran and Integrin α7β1 on Human Hematopoietic Stem Cells

**DOI:** 10.3389/fcell.2021.675240

**Published:** 2021-10-22

**Authors:** Parimala Sonika Godavarthy, Christina B. Walter, Claudia Lengerke, Gerd Klein

**Affiliations:** ^1^Department of Internal Medicine II, Hematology, Oncology, Clinical Immunology and Rheumatology, University Hospital Tübingen, Tübingen, Germany; ^2^Department of Gynecology and Obstetrics, University Hospital Tübingen, Tübingen, Germany

**Keywords:** bone marrow microenvironment, cell adhesion molecule, cell-matrix interactions, extracellular matrix, Lutheran blood group, stem cell niche

## Abstract

In the adult organism, hematopoietic stem and progenitor cells (HSPC) reside in the bone marrow (BM) in specialized hematopoietic stem cell niches of which the extracellular matrix (ECM) is an integral component. Laminins (LM) are a family of heterotrimeric ECM molecules of which mainly family members containing an α4 or α5 chain are expressed in cells from BM niches and involved in HSPC homing and proliferation. Various integrin and non-integrin laminin receptors have been identified and characterized. Among these, the integrins α6β1 and α3β1 were reported to be strongly expressed on human and mouse HSPC. In the present study, we focus on two further specific laminin receptors, namely integrin α7β1 and basal cell adhesion molecule/Lutheran (BCAM/Lu). Using RT-PCR analyses, immunofluorescence staining, immunoblotting and flow cytometry, we show that both are strongly expressed by human lineage-negative CD34 + HSPC. Treatment with function-blocking antibodies against BCAM/Lu neither inhibits the strong adhesive interaction of CD34 + HSPC with LM-511/LM-521 nor the LM-511/LM-521 mediated changes in CD34 + HSPC proliferation, but however, influences the cytokine-induced differentiation of HSPC in colony formation assays. In addition, stromal-derived factor (SDF) 1α-mediated transmigration of CD34 + HSPC through an endothelial cell layer was effectively diminished by BCAM/Lu antibodies, suggesting a direct involvement of BCAM/Lu in the migration process. This study indicates that both laminin receptors newly identified on human CD34 + HSPC should be taken into consideration in future studies.

## Graphical Abstract

Summary of laminin receptors on human CD34 + HSPC.

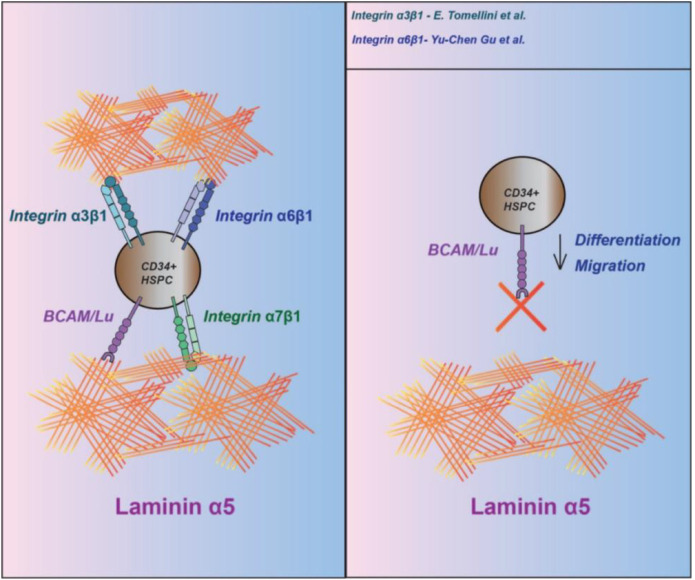

## Highlights

– BCAM/Lu and α7β1 integrin are prominently expressed on human lineage negative CD34 + HSPC.
– CD34 + HSPC strongly attach to laminins containing an α5 chain, but not an α4 chain.
– LM-511 and LM-521 can inhibit proliferation of HSPC, without a direct involvement of BCAM/Lu.
– BCAM/Lu can inhibit the transmigration of CD34 + HSPC through an endothelial barrier.
– BCAM/Lu is involved in the differentiation process of erythroid/myeloid lineage.

## Introduction

The majority of blood cells have a relatively short lifespan and have to be continuously replaced by proliferating and differentiating hematopoietic stem and progenitor cells (HSPC) for the entire life. HSPC reside in specialized locations in the bone marrow known as the hematopoietic stem cell niches, which provide important molecular signals, but also biophysical stimuli to regulate HSPC’ quiescence, self-renewal or differentiation (Morrison and Scadden, 2014; Pinho and Frenette, 2019). A plethora of studies mainly performed in genetically modified mice revealed the existence of two major niches, the vascular niche and the endosteal or osteoblastic niche (Wei and Frenette, 2018). The constituents of the niches include different non-hematopoietic and hematopoietic cell types including mesenchymal stromal cells (MSC; aka mesenchymal stem cells), endothelial cells, adipocytes, fibroblasts, osteoblasts, etc., secreted and membrane-bound chemokines and cytokines and a complex extracellular matrix which despite its known functions for stem cell survival is the least analyzed component of the bone marrow microenvironment (Ahmed and Ffrench-Constant, 2016).

A family of extracellular matrix molecules found in both niches are the laminins which are α–β–γ heterotrimeric molecules consisting of one of five α chains (α1–α5), one of three β chains (β1–β3) and one of three γ chains (γ1–γ3) giving rise to at least 17 different isoforms (Durbeej, 2010). The nomenclature of the laminins reflects the chain composition of the individual isoforms. LM-421, as an example, consists of the α4, the β2 and the γ1 chain. Laminin isoforms containing the α4 or α5 chain are the majorly found isoforms in the bone marrow. They can be detected in basement membranes of different blood vessels (sinusoids, arterioles, or larger vessels) or in adipocytes, in megakaryocytes and in a reticular fiber meshwork in the intersinusoidal spaces (Siler et al., 2000; Susek et al., 2018). On the surface of HSPC, various integrin and non-integrin receptors mediate interactions with the different laminin isoforms. These include the integrin receptors α3β1, α6β1, α7β1, and α6β4, and non-integrin receptors–dystroglycan and BCAM/Lutheran (Durbeej, 2010). The integrin α6β1 is strongly expressed in human and mouse CD34 + HSPC influencing BM homing and engraftment of multilineage hematopoietic stem cells (HSC) (Gu et al., 2003; Qian et al., 2006; Notta et al., 2011). The expression and function of the integrin α6β4 on HSPC has so far not been studied in greater detail (Qian et al., 2006). Controversial data exist about the expression of integrin α3β1 on HSPC in mouse and human studies, where Gu et al. (2003) could not detect α3β1 on freshly isolated murine BM CD34+ cells, but Tomellini et al. (2019) identified α3β1 as a long-term HSC marker on cultured human CD34+ cord blood cells. The integrin α7β1 has so far only been detected on human HSPC by RT-PCR analysis (Schreiber et al., 2009) or shown to be expressed on human bone marrow MSC (Warstat et al., 2010). Using a specialized cDNA array of human HSPC, the expression of dystroglycan on these cells was identified. The results were corroborated by RT-PCR and immunofluorescence analysis, however, a functional involvement of dystroglycan on HSPC is still unresolved (Steidl et al., 2004). Moreover, BCAM/Lutheran which interacts exclusively with laminin isoforms containing the α5 chain has so far only been detected in the erythroid lineage, but not on early HSPC (Southcott et al., 1999; Chasis and Mohandas, 2008).

Since laminins are an integral component of almost all stem cell niches (Krebsbach and Villa-Diaz, 2017), we focused on the expression and function of laminin receptors BCAM/Lutheran and the integrin α7β1 on human HSPC in greater detail in the current study.

## Results

### The BCAM/Lu Receptor on Hematopoietic Stem and Progenitor Cells and Mesenchymal Stromal Cells

**FIGURE 1:**
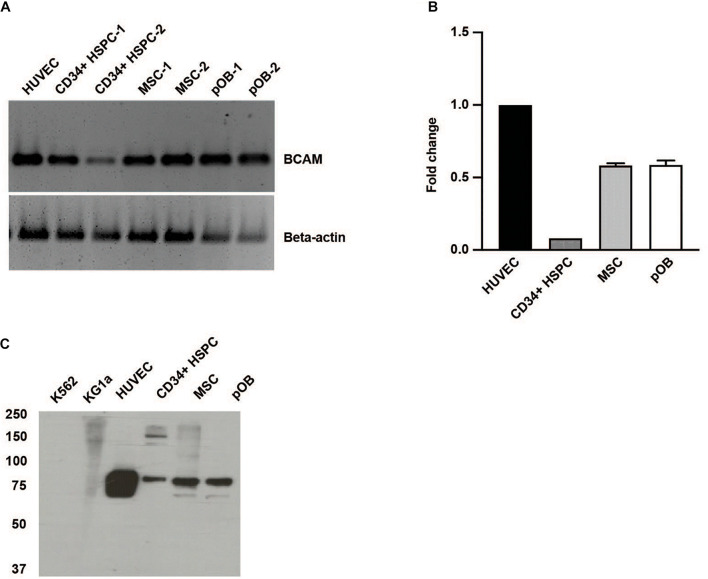
Human HSPC and bone marrow niche cells express BCAM/Lu. **(A)** RT-PCR analysis of CD34 + HSPC, human umbilical vein endothelial cells (HUVEC), bone marrow mesenchymal stromal cells (MSC), and primary osteoblasts (pOB) showing the expression of human BCAM/Lu on all cell types. **(B)** qRT-PCR analysis showing the quantification of expression of BCAM/Lu, and the fold change in expression relative to HUVEC was determined. **(C)** Immunoblotting using a monoclonal antibody against the extracellular domain of BCAM/Lu revealed the expression of BCAM/Lu on HUVEC, CD34 + HSPC, MSC, and pOB, but not on K562 or KG1a cells.

Basal cell adhesion molecule/Lutheran, also known as CD239, exists as two alternatively spliced isoforms of the same gene. They only differ in their cytoplasmic domain resulting in a 78 kDa BCAM and a 85 kDa Lutheran glycoprotein (Daniels, 2009). Using a primer pair recognizing a sequence of the common extracellular domain, we detected BCAM/Lu in CD34 + HSPC, MSC, and also in osteoblasts and endothelial cells used as controls (Figure 1A). Quantitative RT-PCR analysis revealed tenfold more BCAM/Lu transcript in HUVEC cells compared to CD34 + HSPC (Figure 1B). Western blotting also confirmed a much stronger expression of the BCAM/Lu protein in HUVEC in comparison to CD34 + HSPC (Figure 1C), nevertheless demonstrating that BCAM/Lu is a substantial laminin receptor on human CD34 + HSPC. No expression was detected in KG1a and K562 cell lines serving as negative controls (Figure 1C). FACS analysis further revealed the expression of BCAM in THP1 and HUVEC cells, whereas no expression was detected in the K562 cells used as negative control (Supplementary Figure 1). In addition, BCAM was also prominently expressed by the second stem cell type found in the bone marrow, the MSC and their derivatives, the osteoblasts (Figure 1).

**FIGURE 2:**
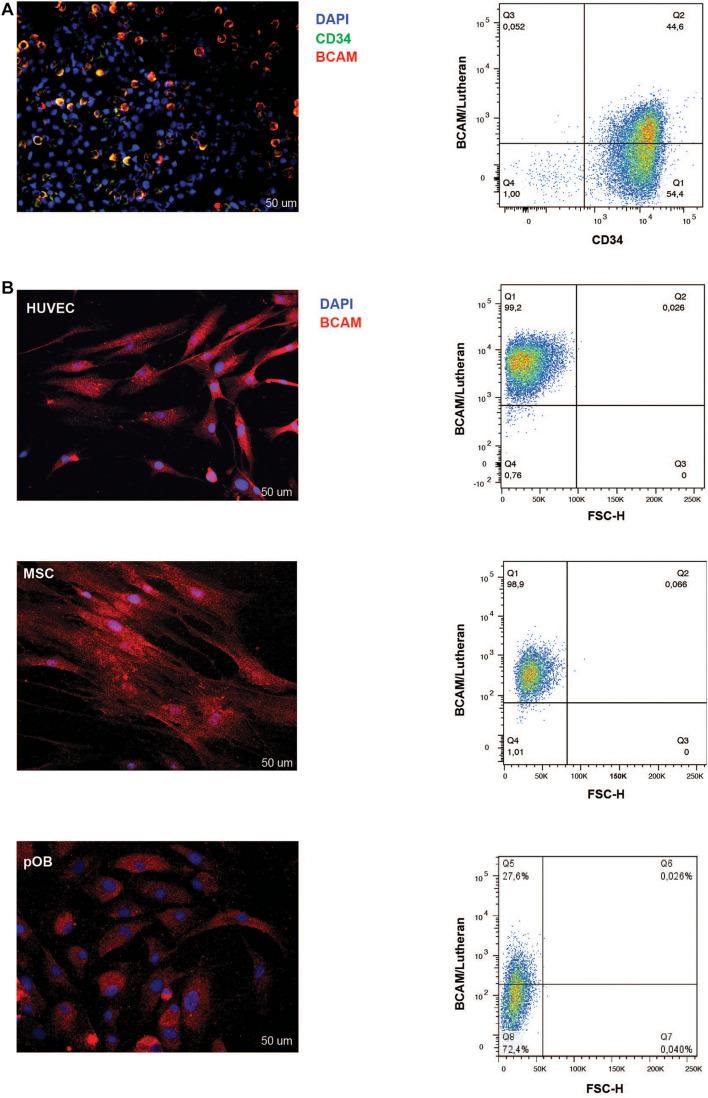
BCAM/Lu expression on CD34+ cells, HUVEC, MSC, and pOB. **(A)** Left: Lineage-negative CBMNC were double-stained with mouse-anti-BCAM/Lu and rabbit-anti-CD34 antibodies followed by Cy3- and Alexa 488-conjugated secondary antibodies, respectively. Cell nuclei of all lineage- cells were counterstained with DAPI (blue signal). The majority of the CD34 + HSPC express BCAM/Lu as seen in the merged immunofluorescence micrograph. Scale bar: 50 μm. Right: FACS blots representing the expression of BCAM/Lu on 50% of the CD34 + HSPCs. **(B)** Left panel: The adherent cells – endothelial, mesenchymal stromal or osteoblastic cells – were labeled with the BCAM/Lu antibody and Cy3-conjugated secondary antibody. Cell nuclei were counterstained with DAPI. Strong immunofluorescence signals were seen on HUVEC and MSC, weaker signals were found on pOB. Scale bar: 50 μm. Right panel: FACS blots representing the expression of BCAM/Lu on HUVEC, MSC, and pOB (top to bottom). Representative plots of three independent experiments.

Immunofluorescence and FACS analysis corroborated these findings. Immunofluorescence staining on isolated lineage-depleted cord blood mononuclear cells indicated the majority of CD34+ cells express BCAM/Lu (Figure 2A). Flow cytometry analysis confirmed that ∼ 45% of CD34+ bone marrow cells show BCAM/Lu expression (Figure 2A). Both techniques further revealed a strong expression of BCAM/Lu in HUVEC and MSC, and detectable, but in comparison weaker expression in osteoblasts (Figure 2B). We analyzed other CAM molecules and interestingly FACS analysis showed expression of only intercellular adhesion molecule-1 (ICAM-1) and not vascular cell adhesion molecule 1 (VCAM-1) on cord blood CD34+ enriched cells (Supplementary Figure 2). The purity of cord blood CD34 + enriched cells was confirmed by FACS analysis of glycophorin A (GPA). Here we could hardly detect any GPA positive cells confirming that BCAM expression in our studies is restricted to cord blood CD34+ cells (Supplementary Figure 3).

### The Integrin α7 Chain on Hematopoietic Stem and Progenitor Cells

**FIGURE 3:**
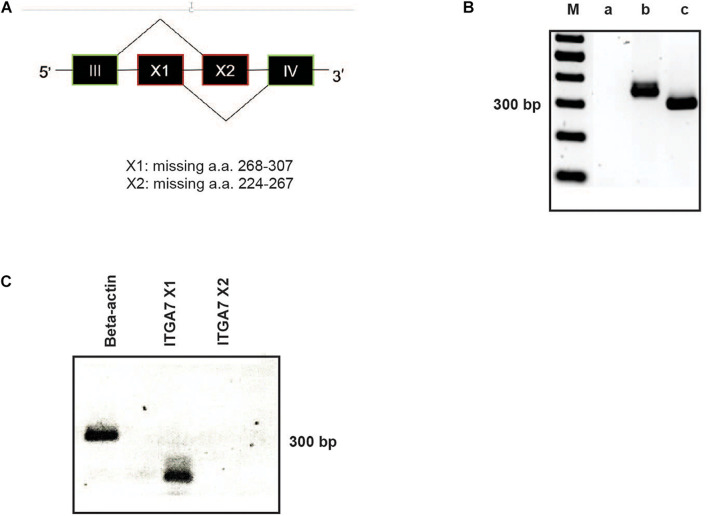
RT-PCR analysis of ITGA7. **(A)** Schematic drawing of the integrin α7X1, X2, and X1X2 isoforms. **(B)** cDNA of isolated CD34 + HSPC was amplified with a specific primer pair for all integrin α7 isoforms (amplification product: 358 bp; lane b). β-actin was amplified as a positive control (317 bp; lane c). Lane a: water control, lane M: 100 bp ladder. **(C)** Using primer pairs specific for the ITGA7 isoform X1 (amplification product: 144 bp) and X2 (232 bp) revealed that CD34 + HSPC transcribe only mRNA for the X1 isoform, no signal for X2 could be detected.

Due to alternatively used exons in the extracellular domain the integrin α7 chain can occur in different isoforms (Collo et al., 1993). In the α7X1 isoform, the amino acids (aa) 268–307 are missing. In the α7X2 isoform, aa 224–267 are deleted, whereas the α7X1X2 isoform contains the entire aa sequence (Figure 3A). Using cDNA of magnetically isolated CD34 + HSPC and a primer pair recognizing a common extracellular sequence, transcription of the integrin α7 chain in these cells could be clearly detected by RT-PCR (Figure 3B). Using X1 and X2 specific primer pairs, the X1 sequence could only be amplified indicating that the X2 or the X1X2 isoforms are not present in human CD34 + HSPC (Figure 3C).

**FIGURE 4:**
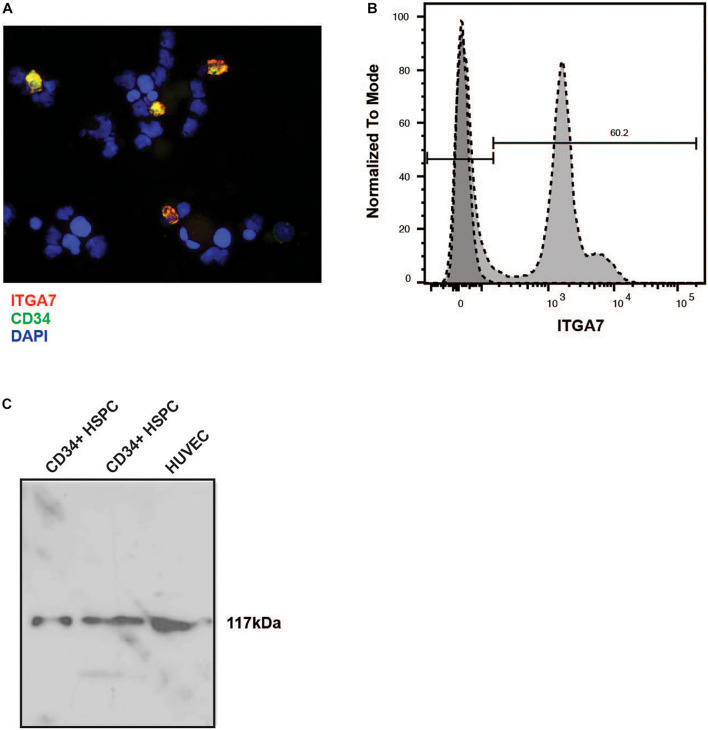
Immunostaining of lineage negative HSPC with the anti-α7 integrin antibody. **(A)** The lineage-negative CBMNC fraction fixed by cytospin centrifugation was labeled with a mouse monoclonal anti-human integrin α7 chain antibody (red) and a rabbit monoclonal anti-human CD34 antibody (green). The cells were counterstained with DAPI (blue). The merged picture shows that all CD34 + HSPC express integrin α7. Representative of three independent experiments. **(B)** Lin- CD34+ cells were labeled with the unconjugated integrin antibody followed by an incubation with FITC-conjugated secondary antibody, and measured by flow cytometry. Representative plot of three independent experiments. **(C)** Two different preparations of lin- CD34+ cell extracts were analyzed by immunoblotting. An extract from HUVEC cells was used as a positive control. In all extracts a band of 117 kb can be detected.

Immunofluorescence labeling of lineage-depleted bone marrow cells revealed that all CD34+ cells of this cell population co-express the laminin receptor integrin α7 (Figure 4A). Flow cytometry confirmed a high expression of integrin α7 in lineage-depleted CD34 + HSPC (Figure 4B). Moreover, Western blot analysis showed a specific band of 117 kDa in CD34+ cells, with HUVEC serving as positive control (Figure 4C).

### Functional Involvement of BCAM/Lu on Hematopoietic Stem and Progenitor Cells

**FIGURE 5:**
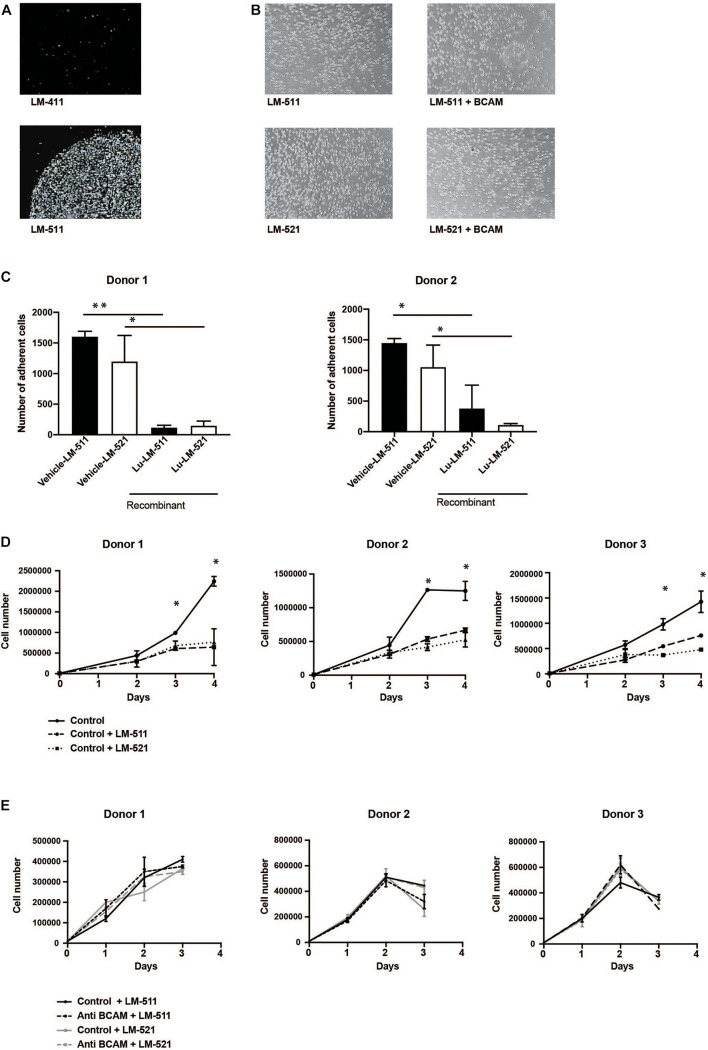
Anti-BCAM/Lu antibodies do not inhibit CD34 + HSPC attachment to LM-511 and LM-521 nor do enhance CD34 + HSPC proliferation in presence of LM-511 and LM-521. **(A)** Cell-matrix adhesion assays were performed with MACS-sorted CD34 + HSPC with LM-411 and LM-511, laminins which were immobilized on the plastic dish. Cell adhesion could only be observed to LM-511, but not to LM-411. **(B)** CD34 + HSPC were allowed to adhere to LM-511, LM-521 in the presence of anti-BCAM/Lu antibodies and without antibodies (control). Representative image of three independent experiments. **(C)** Cell-matrix adhesion assays were performed with MACS-sorted CD34 + HSPC with LM-511 and LM-521, in presence of vehicle and recombinant BCAM/Lu protein. Cell adhesion to LM-511 and LM-521 could be inhibited by recombinant BCAM/Lu protein. **(D)** MACS-sorted CD34+ cells from three donors incubated in serum-free expansion medium with 10 μg/ml LM-511 and LM-521 or without (control) were cultured for three consecutive days. Cells were harvested each day, and the proliferation rate was determined. LM 511 and LM 521 drastically diminished CD34+ cell proliferation. **(E)** Antibodies against BCAM/Lu did not interfere with CD34 + cell proliferation which were cultured for 3 days in serum-free expansion medium in the presence of 10 μg/ml anti-BCAM/Lu antibodies and LM-511 or LM-521. The control without laminin isoforms is not shown in these diagrams. *p < 0.05; **p < 0.005.

A function-blocking antibody against BCAM/Lu (clone #87207) was applied to determine functional interactions of these receptors on HSPC with different laminin isoforms. MACS-isolated CD34 + HSPC strongly attached to LM-511, but not to LM-411 (Figure 5A). These adhesive interactions to LM-511 and LM-521 could not be reduced by treatment with anti-BCAM/Lu antibodies (Figure 5B). However, adhesive interactions of CD34+ cord blood cells with LM-511 and LM-521 were drastically reduced in the presence of recombinant BCAM/Lu protein (Figure 5C).

Culture of CD34 + HSPC in serum-free expansion medium in the presence of LM-511 and LM-521 clearly reduced the proliferation of these cells when compared to control cultures without LM-511/LM-521 (Figure 5D), whereas presence of LM-211 did not affect proliferation (Supplementary Figure 4A). The effect of LM-511 on proliferation of CD34+ cells was also dose-dependent (Supplementary Figure 4B). CFSE proliferation assay also revealed inhibition of proliferation at day 7 as well, explaining the proliferation inhibitory effect to be stable (Supplementary Figure 5A), however, no obvious changes in cell cycle phases were detected by incubation with laminins (Supplementary Figure 5B). These results show that LM-511 and LM-521 are not only strong adhesive substrates for CD34 + HSPC, but also affect their proliferation. However, the inhibitory effect did not seem to be mediated by BCAM/Lu receptors since antibodies against BCAM/Lu did not affect cell proliferation of CD34 + HSPC (Figure 5E).

**FIGURE 6:**
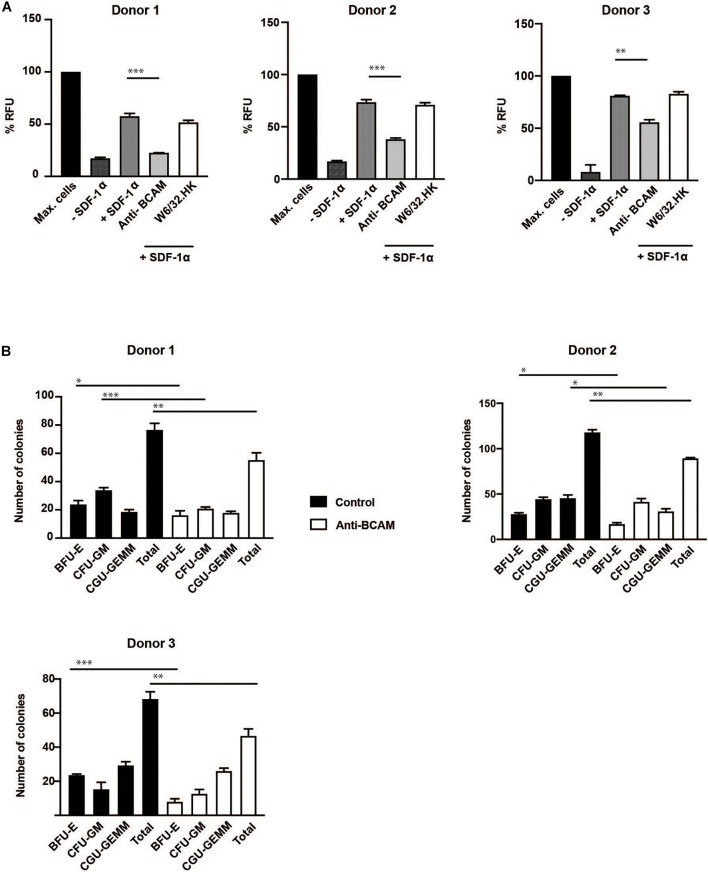
BCAM/Lu regulates transmigration of CD34 + HSPC through endothelial barrier and regulates differentiation. **(A)** MACS-sorted CD34+ cells from three donors were allowed to migrate through an 8 μm polycarbonate membrane pre-coated with a confluent layer of HUVEC. The adherent endothelial cell layer was either cultured without antibodies or treated with 10 μg/ml of BCAM/Lu or W6/32.HK antibodies added to the endothelial cells. The lower chamber was filled with SFEM with or without 100 ng/ml SDF-1α. The lower chamber without the cytokine was the negative control, whereas that containing SDF-1α acted as positive control. The CD34+ cells were allowed to migrate through the membrane for 16 h. The number of migrated cells in the lower chamber was determined measuring the DNA content with the CyQuant. BCAM/Lu antibodies strongly inhibited the SDF-1α induced transmigration in all three donors, whereas the control antibody W6/32.HK showed no inhibitory effect. **(B)** MACS-sorted CD34+ cells from three donors were pre-treated with antibodies against BCAM/Lu for 3 days, and 1000 cells were then plated on methylcellulose. The colonies were scored at day 14. The treatment with antibodies against BCAM/Lu induced lower number of colony formation. *p < 0.05; **p < 0.005; ***p < 0.0005.

Migration assays showed a significant inhibition in transmigration of CD34 + HSPC with BCAM/Lu antibodies. The chemokine SDF-1α strongly promoted transmigration of CD34+ cells through a confluent endothelial cell layer grown on a porous membrane. Pre-treatment of CD34 + HSPC with BCAM/Lu antibodies showed a strong reduction of transmigration toward SDF-1α through endothelial layer in three different donors, whereas pre-treatment with control antibodies did not influence the transmigration rate (Figure 6A). These results implicate an essential role of BCAM/Lu in transmigration through an endothelial cell layer.

Colony formation assay showed a significant decrease in total number of myeloid colonies with BCAM/Lu antibodies, with a decrease in burst forming unit – erythroid (BFU-E) colonies (Figure 6B). These data show an involvement of BCAM/Lu in the differentiation process of CD34 + HSPC into mature cells.

## Discussion

Bone marrow niches support HSC to maintain quiescence and modulate self-renewal and differentiation of these cells into progenitor and mature cells which ultimately migrate out of these niches. Cellular membrane-bound receptors on HSC mediate the interactions between several niche cells and components. Here, we show that two laminin receptors, not analyzed so far in detail on HSPC, are strongly expressed on human CD34 + lineage negative HSPC. Laminin isoforms are expressed both in vascular and endosteal niches, and also in an intricate network between the developing and differentiating hematopoietic cells (Susek et al., 2018).

The laminin isoforms LM-511 and LM-521 have an influence on the proliferation of CD34 + HSPC. This effect was dose-dependent indicating specificity. However, the inhibitory effect did not seem to have an influence on the cell cycle of HSPC. Other laminin isoforms such as LM-211 did not block cell proliferation of CD34 + HSPC indicating that there is no general effect of extracellular matrix components on HSPC proliferation. The isoform LM-332, on the contrary, was shown to have a stimulating effect on MSC proliferation (Hashimoto et al., 2006) clearly indicating that different laminin isoforms have different biological functions.

Basal cell adhesion molecule/Lutheran had been reported to be expressed late in human hematopoietic differentiation, during erythroid development at the orthochromatic erythroblast stage (Southcott et al., 1999; Chasis and Mohandas, 2008). However, autosomal recessive inheritance of the Lutheran null blood group phenotype which has been reported in five individuals showed no obvious associated hematologic pathology (Karamatic Crew et al., 2007). In mice, BCAM/Lu is not expressed in developing or mature red blood cells, and BCAM/Lu-null mice also show no hematological phenotype (Rahuel et al., 2008). Here we demonstrate, using various methods and an antibody raised against a recombinant human BCAM fragment, that BCAM/Lu is expressed at mRNA as well as protein levels at the onset of human hematopoiesis, namely in CD34 + HSPC. In the previous studies, BCAM/Lu expression was only found late during erythroid differentiation but not on early HSPC (Bony et al., 1999; Southcott et al., 1999). In one study, cord blood CD34 + cells were differentiated *in vitro* and analyzed for BCAM expression, but only detected at day 10 after erythroid differentiation, but not day 0 (Southcott et al., 1999). In another study, BCAM expression was not reported in PBMCs. This result could be explained by lack or very low amount of CD34 + cells present in peripheral blood, and hence BCAM/Lu expression was only seen after erythroid differentiation and expansion of the cells. However, whether the differences seen in our and previous studies can be explained by different cell sources or different time points of investigations is still unresolved. However, since also micro-environmental cells of both niches, endothelial cells and MSC, prominently express BCAM/Lu, a vital function of this laminin receptor is anticipated in the human HSC niches. In line, functional data indicate a role for BCAM/Lu in human HSPC differentiation and migration.

Basal cell adhesion molecule/Lutheran is a specific receptor for laminin isoforms containing the laminin α5 chain which is found in basement membranes of bone marrow vascular cells, but also expressed by MSC (Seeger et al., 2015; Susek et al., 2018). Nevertheless, antibodies against BCAM/Lu could not block adhesion of CD34 + HSPC to LM-511 and LM-521. This observation does not rule out that BCAM/Lu is also involved in LM-511 and LM-521 binding since laminin receptors of the integrin family on human HSPC which are also strong binding partners for LM-511 and LM-521 can still mediate the strong adhesive interaction (Gu et al., 2003; Nishiuchi et al., 2006). Whereas the antibody against BCAM/Lu was unable to block attachment of HSPC to LM-511 or LM-521, incubation of both laminin isoforms with the recombinant BCAM/Lu protein blocked adhesion. This could be explained by a direct and complete interaction of the recombinant BCAM/Lu protein with the laminin isoforms leading to steric hindrance of the other laminin receptors on HSPC thus blocking attachment of HSPC to the laminin isoforms.

Basal cell adhesion molecule/Lutheran is not only found on human HSPC or developing and mature erythroid cells, it is also prominently expressed on vascular endothelial cells (Parsons et al., 1995). This might explain why antibodies against BCAM/Lu could affect the cytokine-induced transmigration of HSPC through a BCAM/Lu+ endothelial cell layer. However, a direct effect of antibodies against BCAM/Lu present not only on CD34 + HSPCs but also on endothelial cells cannot be excluded and it would be worth investigating if BCAM/Lu on endothelial cells plays a role in the migration process. It is tempting to speculate that the egress of maturing erythroid cells from erythroblastic islands into the periphery may be facilitated through BCAM/Lu on HSPC and endothelial cells. During embryonic development, migration of HSC from the fetal liver to bone marrow could also be supported by BCAM/Lu since human fetal liver is a rich source of this cellular receptor (Parsons et al., 1995). BCAM/Lu on HSPC can also transfer signals into the hematopoietic cells since antibodies against this receptor can impair the differentiation process of HSPC in colony formation assays. This result indirectly indicates that LM-511 and LM 521 can support the differentiation of HSPC, while inhibiting their proliferation. Whether these opposite effects can be attributed to the presentation of LM-511/LM-521 to HSPC in an extracellular network versus in soluble form is an open question.

The laminin receptor integrin α6 has been identified as a specific marker for human HSC (Notta et al., 2011). Later, it was also shown to be a biomarker for several other stem cell types (Krebsbach and Villa-Diaz, 2017). The observation that only CD34 + cells in the lineage negative bone marrow cell population prominently express the integrin α7 chain supports the notion that this laminin receptor might also be used as a HSC marker. RT-PCR analyses reveal that only the X1, but not the X2 isoform of integrin α7 chain expression on human HSPC. This expression pattern is consistent with the notion that the X1 isoform interacts with LM-511, whereas the X2 isoform only mediate interactions with other laminin isoforms which are hardly found in the bone marrow (Nishiuchi et al., 2006). Unfortunately, no functional data could be obtained for integrin α7 expression on human HSPC, since to the best of our knowledge, no function-blocking antibodies against the human integrin α7 chain are available in the scientific community. Interestingly, an upregulation of the integrin α7 chain could be detected on acute myeloid leukemia (AML) cells with granulocytic sarcoma features. AML cell proliferation could be stimulated with LM-211, an isoform hardly detected in the healthy bone marrow (Kobayashi et al., 2020). It is still unknown if LM-211 can be found in the BM of AML patients and if the AML cells express the X2 isoform interacting with LM-211.

Distinct members of the laminin family were reported to be expressed in a variety of somatic stem cell niches, in putative cancer stem cell niches as well as in the blastocyst stage (Chang et al., 2015; Krebsbach and Villa-Diaz, 2017; Xiang et al., 2020). In the HSC niche, laminin isoforms containing the α4 or α5 chain are prevalent in the extracellular matrix (Siler et al., 2000; Susek et al., 2018). Here we identify two laminin receptors on human HSPC, BCAM/Lu and integrin α7X1β1, which strongly interact with laminins containing the α5 chain. Together with the integrins α6β1 and α3β1, the human HSPC are broadly diversified to interact with these laminin isoforms. Future research will further unravel how these different laminin receptors contribute to HSPC’ adhesion, self-renewal, migration, and proliferation triggered by laminin isoforms in the extracellular matrix, and furthermore explore the relevance of these mechanisms for the pathogenesis of HSPC derived hematologic neoplasms.

## Materials and Methods

### Primary Cells and Cell Lines

All human primary cells were isolated from blood or tissue specimen obtained from hematologically healthy donors after informed consent according to the guidelines of the local ethics committee (reference numbers 005/2012BO2 and 453/2011/BO). For the isolation of HSPC, umbilical cord blood was obtained from the Department of Gynecology and Obstetrics (University Hospital Tübingen). Cord blood mononuclear cells (CBMNC) were isolated by density gradient centrifugation using Histopaque (1.077 g/ml; Sigma-Aldrich, Taufkirchen, Germany) and washed with Dulbecco’s phosphate-buffered saline. CD34 + HSPC were enriched by positive magnetic selection with anti-CD34-conjugated microbeads according to the manufacturer’s instruction (Miltenyi Biotec, Bergisch Gladbach, Germany). To obtain lineage-negative (lin-) stem and progenitor cells, the CBMNC fraction was magnetically labeled with the human lineage cell depletion kit (Miltenyi Biotec) containing biotin-conjugated antibodies against CD2, CD3, CD11b, CD14, CD15, CD16, CD19, CD56, CD123, and CD235a followed by an incubation with anti-biotin microbeads followed by magnetic separation to obtain the unlabeled lineage-negative fraction.

Mesenchymal stromal cells (MSC) were isolated from bone marrow aspirate specimen obtained from the BG Trauma Clinic (University of Tübingen). After Histopaque^®^ enrichment, bone marrow mononuclear cells were seeded in tissue cell-culture flasks in MSC expansion medium containing DMEM low glucose (Lonza, Basel, Switzerland) supplemented with 5% human thrombocyte lysate (Blood Cell Donation Center, University of Tübingen), 5% fresh frozen plasma (TCS Bioscience, Buckingham, United Kingdom), 2 mM L-glutamine (Lonza), 1000 IE heparin sodium salt (Roth, Karlsruhe, Germany) and 25 mM HEPES sodium salt solution (Sigma-Aldrich). After 24 h of culture, the non-attached cells were removed. Adherent MSC were routinely checked for minimal criteria of multipotent MSC (Dominici et al., 2006).

Primary human osteoblasts (pOB) were purified from bone waste of endoprosthesis surgery, obtained from the Department of Orthopedic Surgery (University of Tübingen), according to an established protocol (Staudt et al., 2010). The isolated cells cultured in DMEM (Invitrogen, Karlsruhe, Germany) supplemented with 20% fetal calf serum (FCS), 2% minimum essential medium vitamin solution, 1% fungizone, 1.4 mM β-glycerophosphate and 50 μg/mL ascorbic acid (Sigma Aldrich, Taufkirchen, Germany), were routinely monitored for osteogenic markers. Human umbilical vein endothelial cells (HUVEC) were obtained from PromoCell (Heidelberg, Germany) and cultured in endothelial cell growth medium.

The erythroleukemic cell line K562; the promyeloblastic cell line KG1a and the monocytic cell line THP1 used as negative or positive control cell lines were maintained in RPMI (Invitrogen, Karlsruhe, Germany) supplemented with 10% FCS and 1% Penicillin-Streptomycin.

### Antibodies and Extracellular Matrix Molecules

The mouse monoclonal anti-human BCAM antibody (clone #87207) raised against recombinant human BCAM (amino acids 32–547) was obtained from R&D Systems (Wiesbaden, Germany). This antibody can inhibit the binding of BCAM/Lu to laminin α5 (Kikkawa et al., 2011). A directly conjugated BCAM-PE Cy7 (130-103-915) antibody and the isotype control (130-113-440) were obtained from Miltenyi Biotec (Bergisch Gladbach, Germany). The monoclonal anti-integrin α7 antibody raised against a recombinant fragment corresponding to amino acids 478–578 of human anti-integrin α7 chain was purchased from abcam (Cambridge, United Kingdom). The rabbit monoclonal anti-CD34 antibody (clone EP373Y) was applied (abcam) for indirect immunofluorescence staining. Integrin β1-mediated cell adhesion was blocked with the monoclonal antibody clone 4B4 (Beckmann Coulter, Krefeld, Germany). For control experiments, the undiluted supernatant of the anti-HLA-class I mAb W6/32.HK (inactive variant of W6/32.HL) was used (Barnstable et al., 1978). Directly conjugated FACS antibodies against glycophorin A (GPA) (349119), ICAM-1 (353107), VCAM-1 (305805), ITGA3 (343803) and ITGA6 (313605) were purchased from BioLegend (San Diego, United States). The human recombinant laminin isoforms LM-211, LM-411, LM-511, and LM-521 were purchased from BioLamina (Sundbyberg, Sweden).

### RT-PCR Analysis

Total RNA was isolated from CD34 + HSPC, MSC, pOB, and HUVEC using QIAshredder and the RNeasy^®^ total RNA kit (Qiagen, Hilden, Germany). RNA samples (1 μg) were reversely transcribed using oligo-dT primers and the Superscript III First-Strand Synthesis System (Invitrogen). To amplify the target cDNAs, REDTaq^®^ ReadyMix^TM^ PCR Reaction Mix (Sigma-Aldrich) was used following the instructions of the manufacturer. The forward and reverse primer pairs specific for integrin α7 chain, the X1 and X2 isoforms and BCAM/Lu (see Table 1) were designed using the primer3 program^1^ based on the cDNA sequences published in the GenBank database^2^. As a positive control, a primer pair for β-actin was used. First, cDNA was denatured for 2 min at 94°C, then temperature cycling (35 cycles) was performed: denaturation at 94°C for 30 s, annealing at 60°C for 40 s and elongation at 72°C for 60 s. Final elongation at 72°C for 10 min terminated the temperature cycling. Samples were loaded onto a 2% agarose gel and, after electrophoresis, stained with GelRed^TM^ (Biotium, Hayward, CA, United States). Amplified products were analyzed by exposure under ultraviolet light.

**TABLE 1:** Forward and reverse primer sequences.

**Gene**	**Primer sequence 5′->3′**	**Nucleotide position**	**PCR product size (bp)**	**NCBI accession number**
BCAM/Lutheran	For: AGA GAT GAA CCC AGA GGG CT	654–673	200	NM_005581.4
	Rev: GAT AGT GCA GGG TGA GGT GG	834–853		
GAPDH	For: AAG AAC GTG AAG CTC CCT GA	1523–1542	165	NM_000402.3
	Rev: AAT ATA GGG GAT GGG CTT GG	1668–1687		
Integrin α7 (ITGA7)	For: ATC AAG AGC TTC GGC TAC TCC	1467–1487	358	NM_002206.1
	Rev: GCT TGG GTT CTT CCA GGT TAC	1804–1824		
ITGA7 X1 isoform	For: GGT GGA GCT CTG TGC ACA	903–920	142	NM_001144996.2
	Rev: CCC CGA GTC AAT AGA GAA GCC AAA GTA G	1017–1044		
ITGA7 X2 isoform	For: GCC CCC AAG GCC ATG AAC AAT TTG	782 – 805	232	NM_002206.3
	Rev: CCT AAG TAG CTA TTG AGG GCC	993–1013		
β-actin	For: TCA GAA GGA TTC CTA TGT GGG C	228–249	317	NM_001101.3
	Rev: CCA TCACGA TGC CAG TGG TA	544–525		

### Quantitative RT-PCR Analysis

After reverse transcription, quantitative real-time RT-PCR (qRT-PCR) for BCAM/Lu was performed using FastStart DNA Master^PLUS^ SYBR Green I Reaction Mix (Roche) according to the manufacturer’s instruction on LightCycler^®^ 1.5 (Roche Applied Science). The primer pair for the amplification of BCAM/Lu qRT-PCR was purchased from Qiagen (QuantiTect Primer Assay). After denaturation of cDNA at 95°C for 10 min, temperature cycling (40 cycles) was performed as follows: a denaturation step at 95°C for 10 s, annealing at 58°C for 10 s and an extension step at 72°C for 10 s. Values were normalized to the expression of housekeeping gene glucose 6-phosphate dehydrogenase (GAPDH, Table 1). Cycle thresholds (CT) of the gene of interest (GOI) were compared with those of housekeeping gene (HG) to determine relative expression levels. Relative fold changes between the expression of the GOI in treated and untreated samples were determined by the following equation: fold change = E_GOI_^[ΔCTGOI]^/E_HG_^[ΔCTHG]^, where E = PCR reaction efficacy and [ΔCT GOI] = (C_T  untreated_ – C^T  treated^)_GOI_; [ΔCT HG] = (C_T  untreated_ – C_T  treated_)_HG_.

### Immunofluorescence Staining

For indirect immunofluorescence staining, the adherently growing HUVEC, MSC, and pOB were cultured in 8-well chamber slides (BD BioCoat^TM^ culture slides) until 70–80% confluence, whereas the lineage-negative CBMNC were attached on culture slides by cytospin centrifugation. The cells were fixed on the culture slides by ice-cold methanol for 10 min at −20°C, washed twice with PBS for 5 min and used immediately for the staining procedure. The primary antibodies were diluted in 0.1% BSA/PBS and incubated for at least 1 h in a dark humid chamber. After washing, the fluorochrome-conjugated secondary antibodies diluted in 0.1% BSA/PBS was added and incubated for 1 h. For counterstaining, the secondary antibodies were combined with 1 μg/ml DAPI (4′,6-diamidino-2-phenylindole dihydrochloride; Roche, Mannheim Germany). The cells were washed three times with PBS and embedded in mounting medium (Dako fluorescence mounting medium; Dako, Hamburg). Control staining was performed by omitting the primary antibody. The labeled cells were examined using the fluorescence microscope Zeiss Axiophot (Göttingen, Germany).

### Immunoblotting

Cell lysates were prepared by incubating cell pellets in lysis buffer containing 10 mM Tris–HCl pH 7.4, 150 mM NaCl, 1 mM MgCl_2_, 1 mM CaCl_2_, 1% NP-40, 1% Triton X-100, and a protease inhibitor (cOmplete^TM^, Mini; Merck, Darmstadt, Germany) for 1 h at 4°C. The cells were homogenized by ultrasonification on ice, centrifuged at 13,000 rpm at 4°C and the supernatant was collected. Cell lysates were separated on 10% SDS polyacrylamide gels under non-reducing conditions. PVDF membranes activated in methanol were used for blotting. After transfer, the membranes were blocked with TBS-T buffer (10 mM Tris–HCl pH 8.0, 150 mM NaCl, 0.1% Tween 20) containing 5% milk powder at 37°C for 1 h. The membranes were incubated with specific primary antibodies overnight at 4°C, followed by three washes with TBS-T for 30 min. Bound antibodies were detected with horseradish peroxidase-conjugated secondary anti-mouse antibody and an ECL substrate solution (Merck Millipore).

### Flow Cytometry (FACS)

Expression of BCAM/Lu on human bone marrow cells was studied with an APC-conjugated anti-human BCAM/Lu antibody (clone # 87207; R&D Systems). 5 × 10^5^ cells were pipetted into a round bottomed 96 well plate and washed twice with PFEA buffer (PBS, 2% FCS, 2 mM EDTA, 0.01% sodium azide) at 4°C for 5 min. The F_c_ receptors were blocked with 10% polyglobin solution (Bayer, Leverkusen, Germany) for 10 min. The cells were then directly labeled with the APC-labeled BCAM/Lu antibody for 15 min. For dual color staining, a FITC-coupled anti-human CD34 antibody (Miltenyi Biotec) was added. For the detection of integrin α7 the unconjugated primary antibody was incubated with the lin- CD34 + HSPC cell fraction. After washing, the cells were incubated with FITC-conjugated secondary anti-mouse antibody for 15 min followed by measurement on BD LSR II using FACS Diva software v6.1.3 (BD Biosciences, Heidelberg). The analysis was performed with the FlowJo 7.6.5 Software (FlowJo LLC, Ashland).

### Proliferation Assay

1 × 10^4^ MACS-purified CD34 + HSPC were incubated in duplicates with soluble laminin isoforms (LM-211, LM-411, LM-511 and LM-521), with mouse anti-human BCAM monoclonal antibody or with PBS in 100 μl of serum free expansion medium (SFEM) containing 100 ng/ml stem cell factor, 100 ng/ml Flt-3 ligand, 20 ng/ml interleukin-3 and 20 ng/ml interleukin-6 (StemSpan TM CC100, Stem Cell Technologies, Vancouver, BC, Canada) for 1–3 days in a flat bottom 96 well plate in a humidified atmosphere with 5% CO_2_. On days 1, 2, and 3, cells were counted by Neubauer chamber.

### Cell Attachment

Cell adhesion to laminin isoforms was carried out as described earlier (Klein et al., 1995). Briefly, 100 ng/μl of the laminin solutions were spotted onto plastic dishes and allowed to air-dry at room temperature. Non-specific binding to the plastic dish was prevented by pre-incubation with 1% BSA/PBS. Next, the cells were allowed to attach for 1 h in serum-free medium (supplemented with 1 mM CaCl_2_, 1 mM MgCl_2_ and 25 μM MnCl_2_). For function-blocking experiments, antibodies or isotype controls (MAB003, R&D Systems) were added to the culture medium. For blocking with a recombinant protein, 2 μg of recombinant BCAM/Lu protein (148-BC-100, R&D Systems) were added to the culture medium. After 1 h, non-adherent cells were removed by gently rinsing the dishes with pre-warmed PBS. Specific cell attachment was evaluated under a Zeiss Axiovert microscope. Photographs of representative fields were taken.

### Transmigration Assay

The transwell cell migration assay was performed as follows: HUVEC at a density of 1 × 10^4^ cells in endothelial cell growth medium were plated in the upper chamber of 24-well culture plates with 8 μm polycarbonate membranes (Corning Inc.) that were coated with 0.05% gelatine (Biochrom, Berlin, Germany). The endothelial cells were cultured until a confluent adherent monolayer was reached. Then the adherent cell layer and 5 × 10^4^ CD34 + HSPC could be treated with 10 μg/ml of antibodies in SFEM without cytokines for 30 min at 37°C and 5% CO_2_. The medium was removed from the adherent cell layer and the inserts were placed in a 24 well plate. Then, 5 × 10^4^ CD34 + HSPC suspension treated with or without antibodies was pipetted onto the upper chamber. The lower chamber was filled with SFEM with or without 100 ng/ml stromal cell-derived factor 1α (SDF-1α; PeproTech, Hamburg, Germany). Without SDF-1α the lower chambers were considered as negative control, whereas those containing SDF-1α acted as positive control. The CD34 + HSPC were allowed to migrate for 16 h at 37°C and 5% CO_2_. The number of migrated cells in the lower chamber was determined measuring the DNA content with the CyQuant kit (Invitrogen).

### Colony Forming Unit Assay

1 × 10^4^ purified CD34 + cord blood cells were cultivated with 10 μg/ml of anti-BCAM antibody or PBS as control for 3 days in 100 μl of serum-free expansion medium (SFEM) supplemented with 100 ng/ml Flt-3 ligand, 100 ng/ml stem cell factor (SCF), 20 ng/ml IL-3 (interleukin-3; Miltenyi) and 20 ng/ml IL-6 (interleukin-6). Cells were counted and 3 × 10^3^ cells were suspended in 300 μl of Iscove’s modified Dulbecco’s medium (IMDM) supplemented with 2% fetal bovine serum (Stem Cell Technologies, Vancouver, BC Canada). Cell suspensions were added to 3 ml methylcellulose medium (MethoCult H4434, Stem Cell Technologies) and 1 ml of cell suspension was plated in triplicates on 35-mm petri dishes and cultured in a humidified incubator with 5% CO_2_ at 37°C for 14 days.

Cell aggregates containing >50 cells were scored as single colonies using an inverted microscope (Axiovert 135: Zeiss). Colonies were classified as burst-forming unit – erythroid (BFU-E), colony-forming unit – granulocyte/macrophage (CFU-GM) or colony-forming unit - granulocyte, erythrocyte, macrophage, megakaryocyte (CFU-GEMM) based on their morphology.

### Statistical Analysis

All values are represented as mean ± standard deviation (SD). Statistical analysis was performed with one-way ANOVA or *t*-test using Graph Pad Prism 9 software. Differences were considered to be significant for ^∗^*p* < 0.05 – ^****^p < 0.001.

## Data Availability Statement

The original contributions presented in the study are included in the article/Supplementary Material, further inquiries can be directed to the corresponding author.

## Ethics Statement

The studies involving human participants were reviewed and approved by Ethics Committee of the Medical Faculty of the University of Tübingen. The patients/participants provided their written informed consent to participate in this study.

## Author Contributions

PG: collection and/or assembly of data, data analysis and interpretation, and manuscript writing. CW and CL: provision of study material, data analysis, and interpretation. GK: conception and design, data analysis and interpretation, and manuscript writing. All authors read and approved the manuscript.

## Conflict of Interest

The authors declare that the research was conducted in the absence of any commercial or financial relationships that could be construed as a potential conflict of interest.

## Publisher’s Note

All claims expressed in this article are solely those of the authors and do not necessarily represent those of their affiliated organizations, or those of the publisher, the editors and the reviewers. Any product that may be evaluated in this article, or claim that may be made by its manufacturer, is not guaranteed or endorsed by the publisher.

## Footnotes

1
http://primer3.ut.ee

2
http://www.ncbi.nlm.nih.gov
