# Supplementary material for: The Laminin Receptors Basal Cell Adhesion Molecule/Lutheran and Integrin α7β1 on Human Hematopoietic Stem Cells
Source: Front Cell Dev Biol. 2021 Oct 22;9:675240. doi: 10.3389/fcell.2021.675240 (PMC8570280; doi:10.3389/fcell.2021.675240)
Supplement: Supplementary file 1 [file Data_Sheet_1.PDF]

Supplement:

The laminin receptors BCAM/Lu and integrin α7β1 on human hematopoietic stem cells

Parimala Sonika Godavarthy,[1] Christina B. Walter,[2] Claudia Lengerke,[1] and Gerd Klein[1]

[1] Internal Medicine II, Hematology, Oncology, Clinical Immunology and Rheumatology, Department for Internal Medicine, University Hospital Tübingen ; and [2] Department of Gynecology and Obstetrics; University Hospital Tübingen, Germany

Methods:

1. CFSE staining of proliferating cells

Carboxyfluorescein succinimidyl ester (CFSE) was used to determine cell proliferation of CD34+ HSPC under the influence of laminin isoforms over time. $5x10^4$ CD34+ cord blood cells were labeled in 100 µl of a 5 µM solution of CFSE (Invitrogen) according to the manufacturer's protocol. The success of the labelling was controlled by flow cytometry (LSRII, BD Biosciences, Heidelberg, Germany). After each cell division the signal intensity of the CFSE labeling is diminished by 50%. After the initial staining procedure $1x10^4$ HSPC were incubated in 100 µl of serum-free expansion medium containing StemSpan™ CC100 (Stem Cell Technologies). Either LM-411, LM-511 (10µg/ml each), or PBS as control were added. Cell proliferation was monitored on days 3 and 7 by flow cytometry and data were evaluated using FlowJo software (Tree Star Inc., Ashland, Oregon, USA).

2. Cell cycle analysis

For the analysis of the G0, G1, G2 and S phases of the cell cycle of the CD34+ enriched cord blood cells, the DNA and RNA content of these cells were determined by Hoechst 33342 (DNA) and Pyronin Y (RNA) staining (1) according to an established protocol with slight modifications. $1x10^4$ HSPC were incubated for three days in 100 µl of serum-free expansion medium containing StemSpan™ CC100 (Stem Cell Technologies) together with PBS (control), 10µg/ml LM-411 or 10µg/ml LM-511. After three days of incubation the cells were fixed for 30 min in 20% ethanol. After washing the cells were incubated for 20 min with Hoechst 33342 (2 µg/ml final concentration) and Pyronin Y (4 µg/ml final concentration). Without further washing the cells were analyzed by flow cytometry (LSRII, BD Biosciences).

References:

1. H.A. Crissman, Z. Darzynkiewicz, R.A. Tobey and J.A. Steinkamp: Correlated measurements of DNA, RNA, and protein in individual cells by flow cytometry. *Science*, 228(4705), 1321-1324 (1985) doi: 10.1126/science.2408339.
