# Supplementary figures and images for: The Laminin Receptors Basal Cell Adhesion Molecule/Lutheran and Integrin α7β1 on Human Hematopoietic Stem Cells
Source: Front Cell Dev Biol. 2021 Oct 22;9:675240. doi: 10.3389/fcell.2021.675240 (PMC8570280; doi:10.3389/fcell.2021.675240)

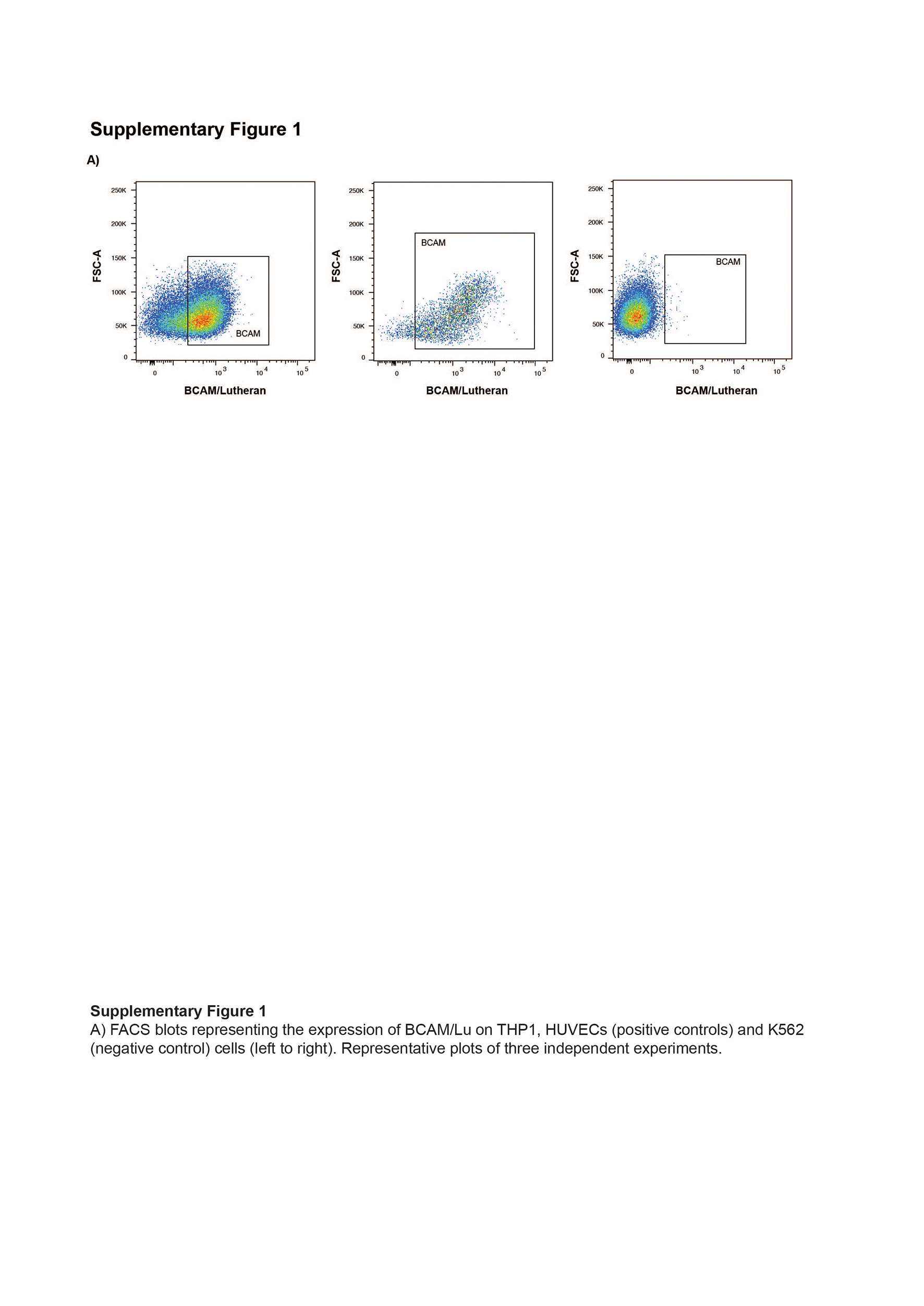

Supplement: Supplementary file 2 [file Image_1.jpeg]

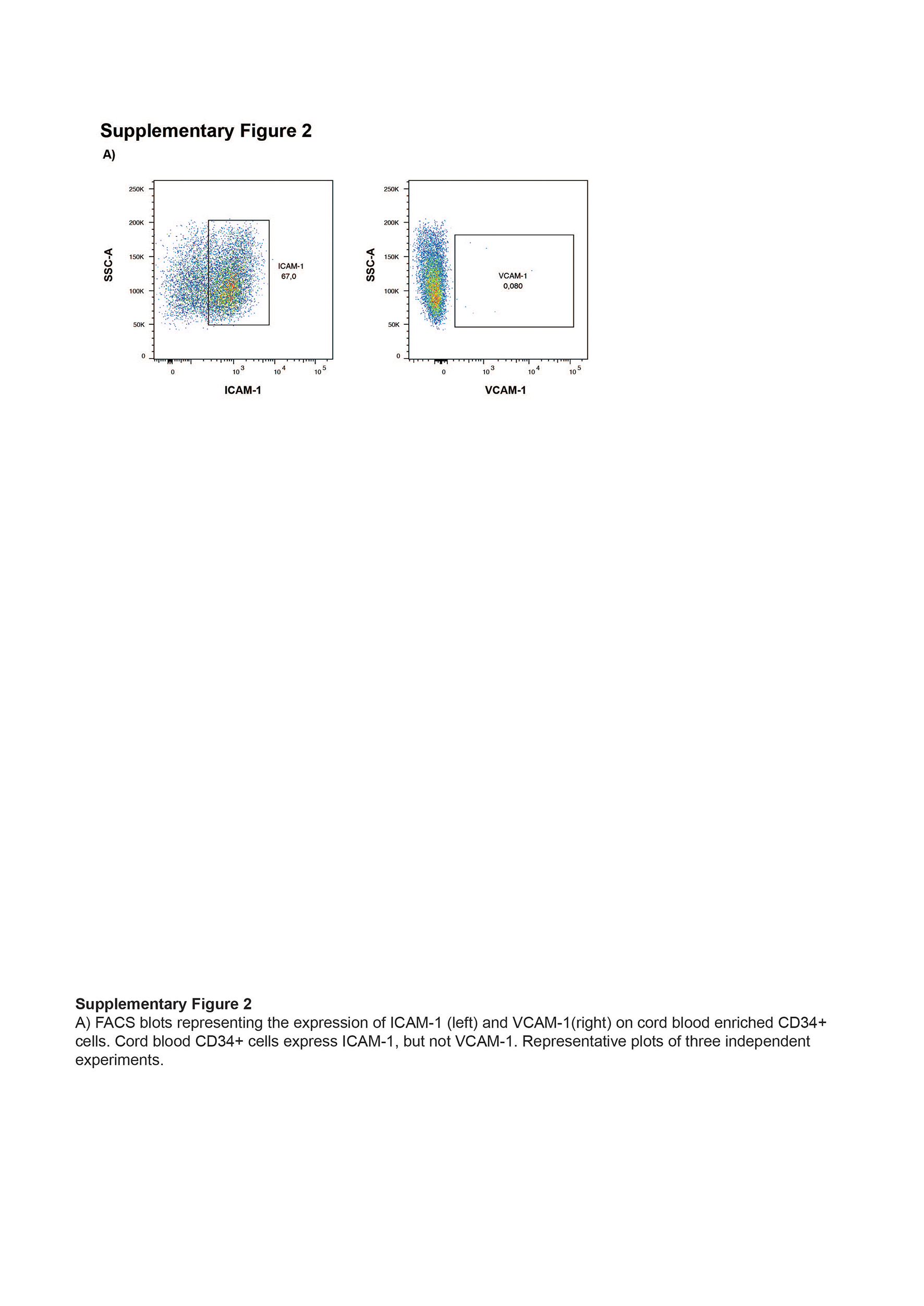

Supplement: Supplementary file 3 [file Image_2.jpeg]

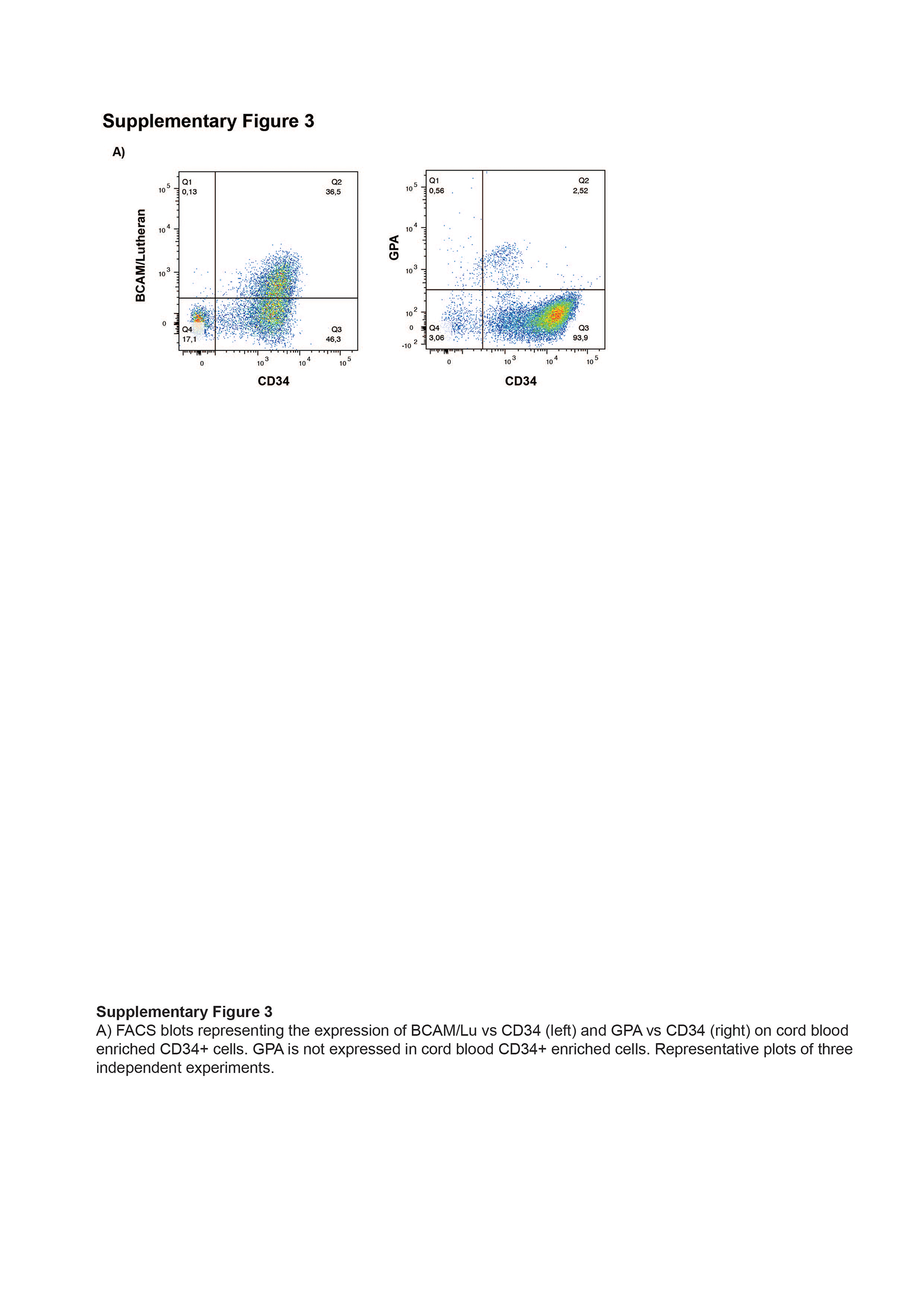

Supplement: Supplementary file 4 [file Image_3.jpeg]

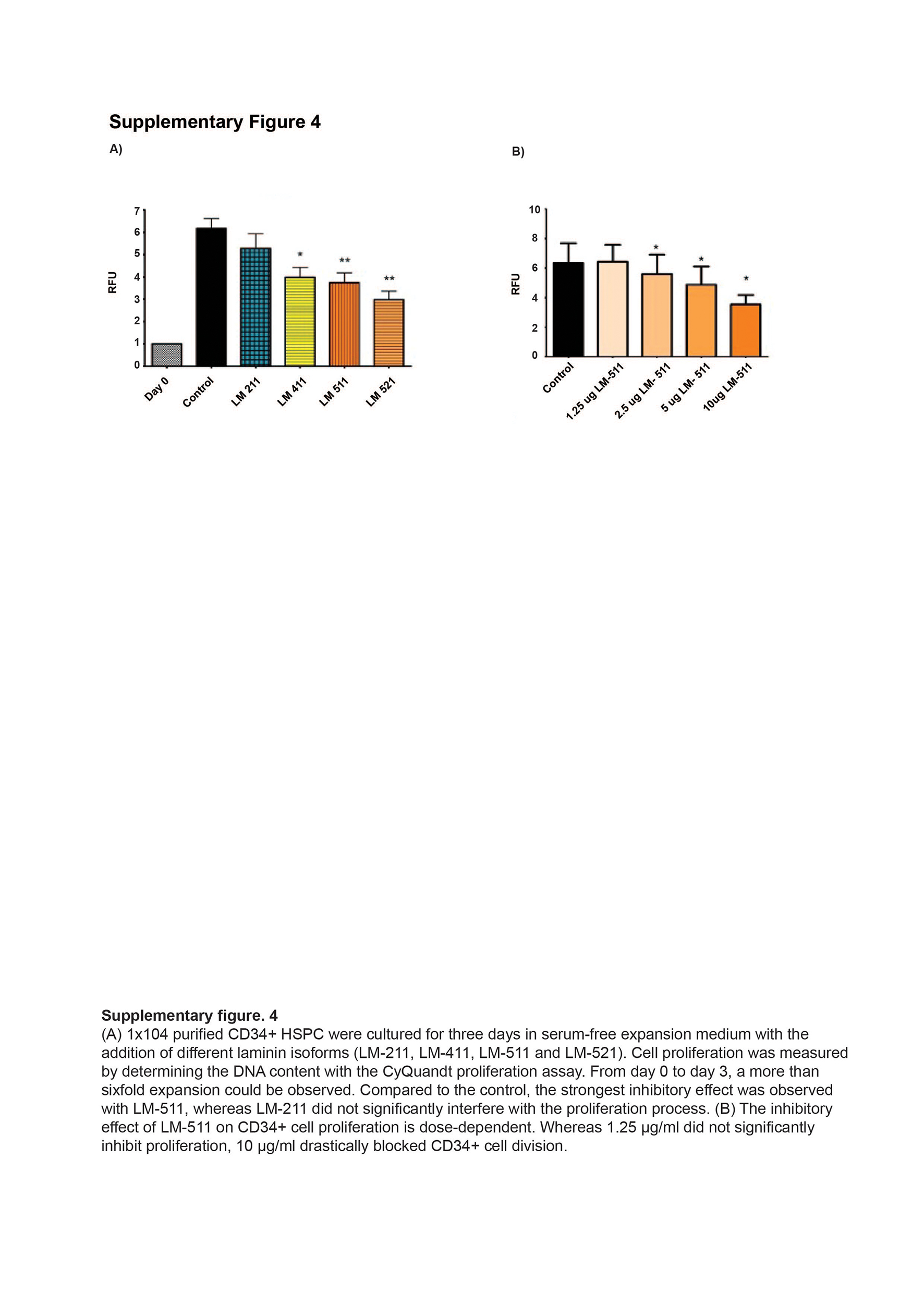

Supplement: Supplementary file 5 [file Image_4.jpeg]

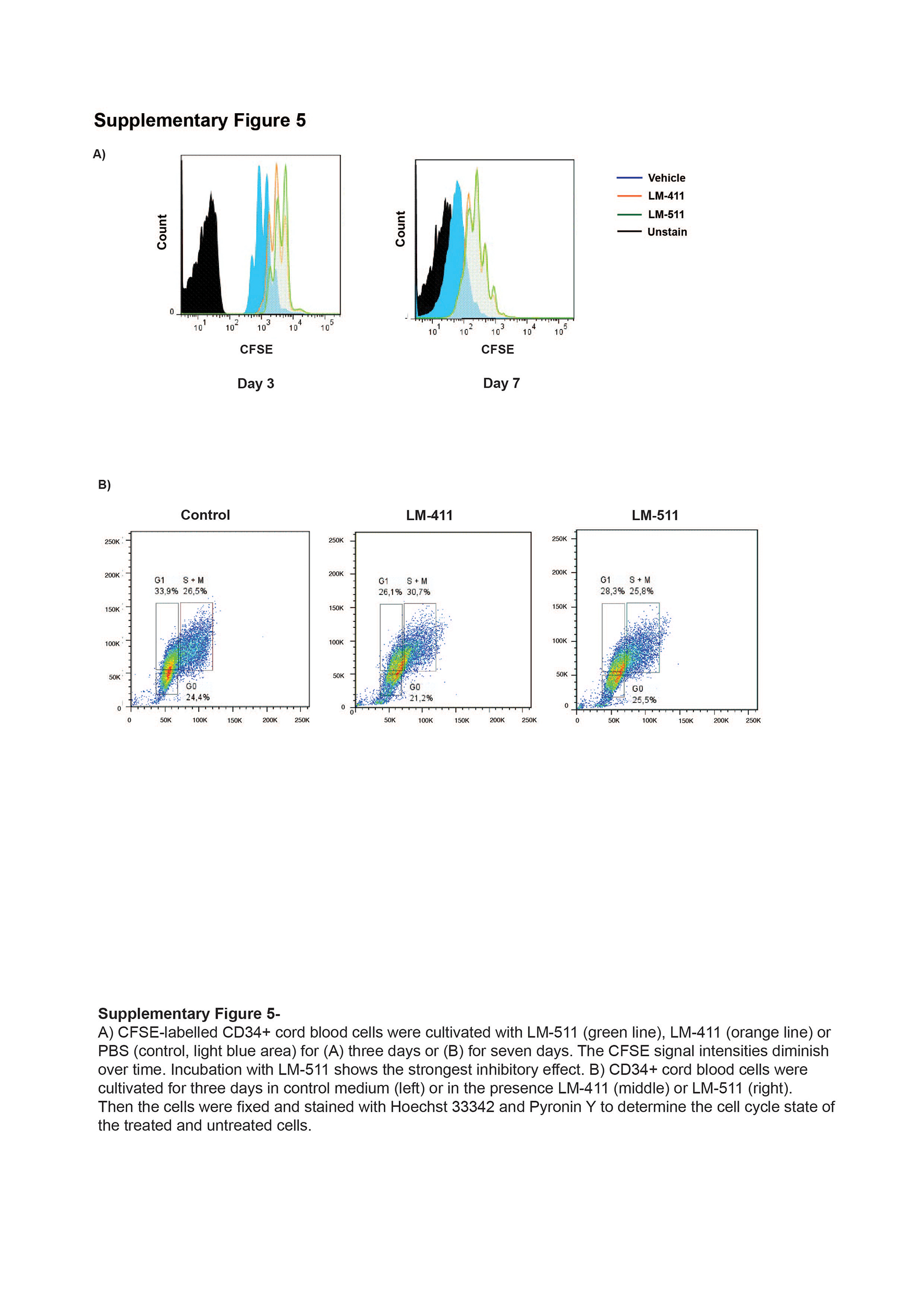

Supplement: Supplementary file 6 [file Image_5.jpeg]
